# Prevalence and clinical correlates of chronic obstructive pulmonary disease in heart failure patients: a cross-sectional study in China

**DOI:** 10.3389/fmed.2025.1477388

**Published:** 2025-02-03

**Authors:** Ailing Zhu, Manman Hu, Dehai Ge, Xiujian Zhang, Jinfeng Zhang, Yangchun Wang, Xin Yao, Junjun Liu

**Affiliations:** ^1^Nanjing Meishan Hospital, Nanjing, China; ^2^The First Affiliated Hospital of Nanjing Medical University, Nanjing, China

**Keywords:** COPD, HF, prevalence, clinical correlates, CCI score, lipid metabolism

## Abstract

**Background:**

Despite chronic obstructive pulmonary disease’s (COPD)'s prevalence in the general populace, its incidence in heart failure (HF) patients is understudied. This study aimed to assess COPD prevalence and clinical associations in Chinese HF patients.

**Methods:**

From the Chinese Heart Failure Study, demographic and clinical details of 2008 HF patients were analyzed. Divided into 233 COPD cases and 1775 non-COPD controls, a multivariable logistic regression identified factors linked to COPD onset in HF, with thorough examination of intergroup clinical differences.

**Results:**

The incidence of COPD in HF individuals was 11.60% (233/2008). The COPD subgroup featured a higher ratio of individuals over 60 and males, alongside lower systolic blood pressure (SBP), body mass index (BMI), higher Charlson Comorbidity Index (CCI) scores, and increased PaCO₂ levels (*p* < 0.05). Type II respiratory failure and right ventricular dysfunction (RVD) were more prevalent in the COPD subgroup (*p* < 0.001). Binary logistic regression, after adjustments, indicated positive associations between COPD and age over 60 (OR = 3.831, 95%CI: 1.085–13.526, *p* = 0.037), male sex (OR = 1.587, 95%CI: 1.032–2.441, *p* = 0.036), higher CCI (OR = 2.214, 95%CI: 1.796–2.729, *p* < 0.001), elevated PaCO_2_ (OR = 1.035, 95%CI: 1.015–1.055, *p* < 0.001), and RVD (OR = 0.605, 95%CI: 0.119–3.063, *p* = 0.544). Inversely, higher SBP (OR = 0.990, 95%CI: 0.982–0.998, *p* = 0.020) and log (triglycerides) (OR = 0.183, 95%CI: 0.064–0.552, *p* = 0.002) were negatively correlated with COPD in HF patients.

**Conclusion:**

In a large cohort of Chinese Heart Failure (HF) patients, our study revealed a notable COPD prevalence. Key risk factors included age, sex, elevated PaCO_2_, CCI score, and right heart failure, while higher SBP and triglyceride levels offered protection. These insights lay groundwork for probing disease mechanisms and therapeutic approaches.

## 1 Introduction

Chronic Obstructive Pulmonary Disease (COPD) is the fourth-leading cause of mortality worldwide, contributing significantly to the rising social and economic burden (1, 2). Global estimates of the number of people with COPD aged 30–79 years old are 391.9 million, based on the Global Initiative for Chronic Obstructive Lung Disease (GOLD) criteria (3). Approximately 99.9 million people in China are affected by a prevalence rate of 8.6% of spirometry-defined COPD (4). COPD frequently coexists with cardiovascular diseases, leading to more severe outcomes than either condition alone (5, 6). The three most common cardiac comorbidities of COPD are heart failure, ischemic heart disease, and atrial fibrillation (7). Both COPD and HF are linked to increased mortality, increased healthcare expenses, and there is a pressing need for better treatment approaches.

The association between COPD and HF has significant systemic implications, characterized by a chronic and progressive course that impairs exercise tolerance (8). Emerging research points to modifications in vascular molecular processes as potential initiators of pulmonary emphysema, with a particular emphasis on the significance of the NO-sGC-cGMP axis. This pathway is now recognized as a key player in both the pathophysiology of the condition and its inherent restorative capabilities (9). Several significant risk factors for COPD have been found, including male gender, smoking, body mass index (BMI), biomass exposure, and occupational exposure to dust or smoke (2, 3). Risk factors for COPD and HF are similar and include low-grade systemic inflammation, smoking, and advanced age (7). Despite this, there has been limited research on identifying biomarkers associated with COPD, particularly in HF patients. There is a lot of data to support the theory that dyslipidemia contributes significantly to the development of COPD (10). In an Indian population study, components of metabolic syndrome (MetS) such as BMI, systolic blood pressure (SBP), triglycerides (TG), high-density lipoprotein (HDL) cholesterol, and fasting blood glucose (FBG) were significantly correlated with COPD (11). Further research has also shown that people with MetS have higher odds of high blood pressure (BP), high TG, poor HDL cholesterol, abdominal obesity, and other COPD risk factors (12, 13). Few studies have focused on lipids as biomarkers for COPD in HF patients.

Most COPD incidence studies emphasize age, sex, smoking, and BMI, rarely addressing blood counts, biochemistry, and blood gas analysis impacts. And there is a dearth of epidemiological data on the risk factors for COPD in HF patients, particularly among the Chinese population. The principal objectives of this study were twofold: firstly, to ascertain the prevalence of COPD in HF inpatients; and secondly, to evaluate the relationship between COPD and HF in Chinese individuals, encompassing demographic characteristics, biochemical indicators, CCI scores, and other pertinent factors. By elucidating these relationships, we aim to inform and enhance the development of more targeted therapeutic strategies for patients with coexisting COPD and HF.

## 2 Methods

### 2.1 Data source

The study population was obtained from an open-source online database of patients with HF in China, accessed via the PhysioNet platform (14). A total of 2008 inpatients were recruited at Zigong Fourth People’s Hospital between December 2016 and June 2019. The research design and database information have been described in detail (15). The study protocol and methodologies were accepted by the Ethics Committee of Zigong Fourth People’s Hospital (approval number: 2020-010). The study adhered to the principles outlined in the Declaration of Helsinki.

### 2.2 Study population

Patients admitted to the hospital with a diagnosis of heart failure were enrolled in the study. Electronic healthcare records of consecutive patients diagnosed with HF were reviewed (15). The database included all types of HF—including acute, chronic, left-sided, right-sided, and mixed presentations diagnosed in accordance with the 2016 European Society of Cardiology (ESC) criteria (16).

1. Clinical signs and symptoms of heart failure (HF): fatigue, poor exercise tolerance, delayed recovery after exercise, ankle swelling, and dyspnea, especially orthopnea and nocturnal dyspnea. Clinical indicators of HF include third heart sound (S3 gallop), hepatojugular reflux, high jugular venous pressure, and lateral apical impulse displacement.
2. Biomarker Levels: >35 pg./mL elevated BNP or > 125 pg./mL NT-proBNP.
3. Cardiac Structural/Functional Changes: Objective evidence supporting HF diagnosis.
4. Diagnostic Confirmation: For unclear cases, stress tests or invasive assessment of LV filling pressures confirms HF.

Heart failure can be categorized into left-sided, right-sided, and biventricular types. Left-sided failure includes systolic (reduced ejection fraction) and diastolic (preserved ejection fraction) forms, while right-sided failure often results from left-sided issues or conditions affecting the right ventricle directly, and biventricular failure impacts both ventricles simultaneously (16). We utilized various echocardiographic parameters to group and evaluate heart failure patients, including left ventricular ejection fraction (LVEF), mitral valve flow E/A ratio, tricuspid annular plane systolic excursion (TAPSE), and right ventricular systolic peak velocity (RV S′), among others. However, the source data lacked information on diastolic and systolic heart failure (15). Therefore, our analysis is based on classifications of left heart failure, right heart failure, and global heart failure.

### 2.3 Definitions of other key parameters

Type II respiratory failure, also known as hypercapnic respiratory failure, is defined by the inability of the respiratory system to adequately eliminate carbon dioxide, resulting in elevated arterial carbon dioxide levels (PaCO_2_ ≥ 45 mmHg) along with hypoxemia (17).

The fraction of inspired oxygen (FiO2) denotes the proportion of oxygen in the gas mixture that the patient breathes in, typically expressed as a fraction or percentage (18).

### 2.4 Sociodemographic characteristics and assessments of comorbidities

Demographics, initial clinical parameters, comorbidities, laboratory results, medicines, and outcomes were the six categories that made up the dataset. A historical case of congestive heart failure, myocardial infarction, dementia, Parkinson’s disease, peripheral vascular disease, cerebrovascular disease, peptic ulcer disease, connective tissue disease, diabetes, hemiplegia, and leukemia were among the comorbidities that were assessed. By reviewing electronic medical records (EMRs) and referencing disease codes rather than the gold standard for lung function, the study population was divided into two groups based on the presence or absence of COPD. Signs of emphysema and alveolar changes were evident on CT scans for the majority of patients, thereby supporting the diagnosis of COPD. The weighted scores of the comorbid conditions listed above were added up to create the Charlson Comorbidity Index (CCI), which considers the number and severity of comorbid diseases, assesses the risk of mortality (19, 41).

### 2.5 Blood samples

Laboratory findings were obtained on the first day of hospital admission, following an eight-hour fast and included the following parameters: serum uric acid, creatinine, glomerular filtration rate (eGFR), cystatin C, hemoglobin, platelet, D-dimer, high-sensitivity troponin, Glutamic-oxaloacetic transaminase (GOT), Gamma-glutamic transferase (GGT), total cholesterol, low-density lipoprotein (LDL) cholesterol, triglycerides, HDL cholesterol, blood pH, and partial pressure of carbon dioxide (PaCO₂).

### 2.6 Statistical analysis

The statistical software SPSS 26.0 was used for the analyses. The normality of continuous variables was evaluated using the Kolmogorov–Smirnov technique. The means ± standard deviation (SD) of continuous variables with a normal distribution were displayed, and the independent samples *T*-test was used to compare groups. Log transformation was utilized for variables that exhibit non-normal distributions, helping to normalize the data and stabilize variance for improved statistical analysis. The categorical variables were summed up as percentages and frequencies, and the χ2 test was used to compare groups. Spearman correlation coefficients were used to analyze relationships between biological markers and clinical and demographic variables. To eliminate variables with variance inflation factor (VIF) values greater than five, we ran a VIF screening (19). The factors impacting the development of COPD in the HF population were examined using binary logistic regression analysis, which included variables with a *p* value <0.05 in the univariate analysis that were highly clinically relevant. Each two-tailed statistical test had a significance level of *p* < 0.05.

## 3 Results

### 3.1 Prevalence and factors associated with COPD

All 2008 HF patients were enrolled in the study; 233 of these patients also had concurrent COPD diagnoses, for an overall rate of 11.60%. Table 1 presents a comprehensive comparison of clinical factors and sociodemographic information between the COPD and non-COPD groups. The COPD group exhibited a higher frequency of older males than the non-COPD groups. Additionally, the COPD group showed significantly lower SBP (*t* = 2.04, *p* = 0.042) and body mass index (BMI) (*t* = 2.20, *p* = 0.028), as well as higher Charlson Comorbidity Index (CCI) scores (*t* = −12.75, *p* < 0.001) and inspired oxygen index (*t* = −3.49, p < 0.001). The incidence of type II respiratory failure (*χ^2^* = 25.51, *p* < 0.001) and right heart failure (*χ^2^* = 14.421, *p* = 0.001) was significantly higher in the COPD group. Moreover, our analysis showed higher PaCO_2_ (*t* = −3.91, <0.001) and lower log (triglyceride) (*t* = 3.95, *p* < 0.001) in the COPD group (Table 1).

**Table 1:** Characteristics of HF patients with and without COPD.

Variables	All patients (*n* = 2008)	Non COPD (*n* = 1775)	COPD (*n* = 233)	t/χ2	*p*
Age, year				8.163	0.004
≥60	1830 (91.1%)	1,606 (90.5%)	224 (96.1%)		
21–59	178 (8.9%)	169 (9.5%)	9 (3.9%)		
Sex				22.96	<0.001
Male	845 (42.1%)	713 (40.2%)	132 (56.7%)		
Female	1,163 (57.9%)	1,062 (59.8%)	101 (43.3%)		
Body temperature (°C)	36.42 ± 0.44	36.42 ± 0.44	36.39 ± 0.43	0.80	0.424
Pulse (bpm)	85.24 ± 21.54	85.34 ± 21.81	84.45 ± 19.39	0.59	0.554
Respiration (bpm)	19.09 ± 1.74	19.06 ± 1.75	19.26 ± 1.61	−1.65	0.100
SBP (mmHg)	131.06 ± 24.74	131.46 ± 24.99	127.95 ± 22.55	2.04	0.042
DBP (mmHg)	76.57 ± 14.46	76.69 ± 14.67	75.66 ± 12.76	1.02	0.306
BMI (kg/m^2^)	21.30 ± 3.81	21.37 ± 3.80	20.79 ± 3.81	2.20	0.028
CCI score	1.86 ± 0.96	1.77 ± 0.93	2.59 ± 0.86	−12.75	<0.001
FiO_2_ (%)	32.67 ± 4.76	32.54 ± 4.65	33.69 ± 5.44	−3.49	<0.001
LVEF (%)	50.68 ± 13.22	50.84 ± 13.18	49.25 ± 13.63	0.90	0.366
LVEDD (mm)	53.11 ± 10.92	53.15 ± 10.99	52.82 ± 10.42	0.35	0.730
Creatinine (μmol/L)	9.57 ± 5.55	9.60 ± 5.64	9.34 ± 4.81	0.67	0.506
Uric acid (μmol/L)	483.02 ± 169.66	483.38 ± 169.26	480.32 ± 172.94	0.26	0.797
eGFR (ml/min /1.73m^2^)	68.66 ± 36.61	68.79 ± 37.30	67.75 ± 30.96	0.46	0.643
Cystatin (mg/l)	1.84 ± 0.95	1.85 ± 0.96	1.78 ± 0.89	1.06	0.289
Hemoglobin (g/l)	115.07 ± 24.53	114.70 ± 24.82	117.83 ± 22.08	−1.82	0.068
Platelet (10^9^/L)	145.15 ± 64.92	146.43 ± 66.55	135.50 ± 50.11	2.41	0.016
D-dimer (mg/L)	2.45 ± 5.33	2.41 ± 5.31	2.70 ± 5.49	−0.72	0.469
Log (hs-TnT) (pg/ml)	−0.18 ± 0.97	−0.20 ± 0.97	−0.09 ± 0.94	−1.55	0.122
Albumin (g/L)	36.52 ± 4.98	36.59 ± 5.01	35.98 ± 4.67	1.72	0.085
ALT (U/L)	61.07 ± 68.50	60.94 ± 68.97	62.05 ± 65.01	−0.23	0.819
AST (U/L)	53.97 ± 204.02	64.23 ± 312.32	55.60 ± 141.00	0.39	0.696
TC (mmol/L)	3.72 ± 1.09	3.73 ± 1.09	3.66 ± 1.05	0.84	0.398
LDL (mmol/L)	1.86 ± 0.76	1.87 ± 0.76	1.80 ± 0.77	1.27	0.205
Log (Triglyceride) (mmol/L)	0.00 ± 0.22	0.01 ± 0.22	−0.06 ± 0.20	3.95	<0.001
HDL (mmol/L)	1.10 ± 0.35	1.10 ± 0.35	1.13 ± 0.35	−1.14	0.254
PH	7.41 ± 0.07	7.41 ± 0.08	7.41 ± 0.07	0.85	0.396
PaCO_2_ (mmHg)	35.80 ± 9.56	35.18 ± 8.95	39.00 ± 11.75	−3.91	<0.001
Type II respiratory failure				25.51	<0.001
Yes	114 (5.7%)	84 (4.7%)	30 (12.9%)		
No	1894 (94.3%)	1,691 (95.3%)	203 (87.1%)		
NYHA classification (%)				1.134	0.567
II	353 (17.6%)	310 (17.5%)	43 (18.5%)		
III	1,039 (51.7%)	926 (52.2%)	113 (48.5%)		
IV	616 (30.7%)	539 (30.3%)	77 (33.0%)		
Type of heart failure				14.421	0.001
Left	477 (23.8%)	399 (22.5%)	78 (33.5%)		
Right	51 (2.5%)	44 (2.5%)	7 (3.0%)		
Both	1,480 (73.7%)	1,332 (75.0%)	148 (63.5%)		

SBP, systolic blood pressure; DPB, diastolic blood pressure; BMI, body mass index; CCI score, Charlson Comorbidity Index score; FiO_2_, fraction of inspired oxygen; LVEF, left ventricular ejection fractions; LVEDD, left ventricular end-diastolic diameter; eGFR, estimated glomerular filtration rate; ALT, glutamyl transpeptidase; AST, aspartate aminotransferase; HDL, high density lipoprotein; LDL, low density lipoprotein; NYHA, New York heart association.

### 3.2 Independent predictors for the risk of COPD in HF

The study employed binary logistic regression analysis to identify independent determinants of COPD risk in individuals with heart failure. Age, sex, SBP, BMI, CCI score, inspired oxygen index, type II respiratory failure, heart failure type, PaCO_2_, and triglyceride were among the factors. The results of the analysis showed that the following factors had a positive correlation with COPD: age (OR = 3.831, 95% CI 1.085–13.526, *p* = 0.037); male sex (OR = 1.587, 95% CI 1.032–2.441, *p* = 0.036); CCI score (OR = 2.214, 95% CI 1.796–2.729, *p* < 0.001); PaCO_2_ (OR = 1.035, 95%CI 1.015–1.055, *p* < 0.001); the right heart failure (RHF) group (OR = 2.344, 95% CI 1.504–3.653, *p* < 0.001) (compared to the left heart failure group). On the other hand, in HF patients, higher log (triglyceride) level (OR = 0.183, 95%CI 0.064–0.552, *p* = 0.002) and high SBP (OR = 0.990, 95%CI: 0.982–0.998, *p* = 0.020) were found to be negatively correlated with COPD (Table 2).

**Table 2:** Related factors of COPD in patients with HF based on binary logistic regression.

Variables	OR	95%CI	*p*
Age (year)	3.831	1.085–13.526	0.037
Sex			
Female	1 (reference)		
Male	1.587	1.032–2.441	0.036
SBP (mmHg)	0.990	0.982–0.998	0.020
BMI (kg/m^2^)	0.973	0.919–1.029	0.335
CCI score	2.214	1.796–2.729	<0.001
inspired oxygen index	1.021	0.992–1.050	0.167
Hemoglobin (g/l)	1.008	0.999–1.018	0.096
Log (hs-TnT) (pg/ml)	1.016	0.817–1.263	0.884
Albumin (g/L)	0.979	0.934–1.026	0.379
Log (Triglyceride)(mmol/L)	0.183	0.064–0.552	0.002
PaCO_2_ (mmHg)	1.035	1.015–1.055	<0.001
Type II respiratory failure	1.468	0.751–2.869	0.261
Type of heart failure			
Left	1 (reference)		
Right	2.344	1.504–3.653	<0.001
Both	1.653	0.327–8.372	0.544

SBP, systolic blood pressure; BMI, body mass index; CCI score, Charlson Comorbidity Index score.

## 4 Discussion

This cross-sectional study is the first that we are aware of that estimates the prevalence and clinical correlates of COPD in a sizable sample of HF Chinese patients. Our findings indicate that the prevalence of COPD in this population is 11.60%. The finding is consistent with a previous study (20). Pellicori et al. reported a prevalence range of 10 to 20% for COPD among patients with HF in both observational studies and randomized controlled trials. This discrepancy may suggest that COPD is often underdiagnosed by cardiologists or that HF exerts pulmonary effects that resemble those of COPD, potentially leading to overdiagnosis (21). Our study also discovered that COPD patients with HF had significantly higher CCI scores, and PaCO_2_, but lower triglycerides and SBP compared to patients with HF alone (*p* < 0.05). Moreover, the COPD group exhibited an increased rate of right heart failure.

According to our research, males over 60 were more likely than women in the same age group to have COPD, which is consistent with previous research findings (22–27). In China, multiple studies have reported that age is a significant factor in the prevalence of COPD (23–25). Specifically, the risk of COPD doubles for every additional 10 years of age among adults aged 40 and above. Furthermore, the decrease in forced expiratory volume in one second (FEV1) accelerates more significantly in older individuals than in younger individuals (28). The higher incidence of COPD in men is likely related to the higher prevalence of smoking among men, which is a proven independent risk factor for COPD. In addition, higher levels of estrogen in women may offer a protective effect, reducing the incidence of persistent airflow limitation by promoting alveolar regeneration and maintaining alveolar structure (29).

There has not been much research done on the correlation between blood pressure and COPD in heart failure patients. Although hypertension does not directly cause COPD, it may indirectly increase the risk by worsening lung function via systemic inflammation, oxidative stress, vascular issues, and autonomic imbalance (13). In contrast to the findings of an earlier article (11), the present findings indicate a higher frequency of COPD in HF with a lower SBP. However, in the previous study, there was no statistically significant change in SBP between the groups with COPD and those without. A monotonic relationship was found by Rao et al. between SBP and the risk of several cardiovascular endpoints, with COPD patients having the lowest risk at SBP <120 mmHg (30). It’s possible that variations in research populations and endpoints are the cause of the disparity between our findings and those of earlier investigations. To confirm that these results are coherent, more research is needed. These findings imply that, in order to prevent negative consequences, blood pressure in HF patients should be kept within an ideal range.

In our study, patients with HF who had higher CCI scores had an increased chance of developing COPD. These results are in line with earlier research (31, 32). Patients with a higher CCI score exhibited more severe lung function impairment and a higher mMRC score in comparison to those with a lower CCI score (31), all of which are crucial conditions for the development of COPD. The comorbidities observed may share common risk factors, such as systemic inflammation, vascular endothelial dysfunction, and hypoxia, which have all been associated with an increased incidence of COPD and may act as risk factors for its development (33).

It is commonly recognized that dyslipidemia contributes significantly to the advancement of COPD (10). The leading role of lipid metabolism in the lung extends beyond serving as a structural or energy substrate; it is also crucial in the immune defense of the lung (10). In COPD, lipid metabolic pathways and molecules undergo significant changes, which can subsequently influence the functions of specific cells—such as the production of inflammatory mediators, immune regulation, and cell death—thereby contributing to the progression of the disease (34). In our study, low triglyceride levels were identified as a risk factor for COPD (OR = 0.183). However, this finding was not statistically significant in an Indian population (11). This may be attributed to the study focusing on a specific subgroup of heart failure patients who had a poorer nutritional status compared to the general population. Previous studies have consistently demonstrated a positive correlation between a low BMI and the prevalence of COPD. This association has been identified as a potential risk factor for COPD and an independent indicator of poor prognosis (22, 24–27). These patients typically exhibit poorer lung function compared to those with a higher BMI, but similar to our study, this association did not reach statistical significance (10) and warrants further investigation in our specific group.

Correlation analysis in the study shows that the incidence of COPD patients was positively correlated with higher PaCO_2_ levels. This finding aligns with a previous study (35), which demonstrated that subjects with a higher level of PaCO_2_ levels had lower FEV1% levels. In individuals with severe COPD, expiratory flow limitation and dynamic lung hyperinflation can lead to increased hypercapnia (36). Higher PaCO_2_ levels could therefore be a sign of severe COPD. Furthermore, restrictive ventilatory abnormalities, which lower vital capacity by replacing oxygen in the lungs with blood or interstitial fluid and cause hypercapnia, are frequently seen in heart failure patients (37). It is important to ask why COPD patients are more likely than CHF patients to retain CO2. Submissive hypercapnia, an adaptive brain mechanism to conserve breathing effort when recovering normocapnia is difficult or impractical, may be the cause of this phenomenon (38). Moreover, a higher risk of long-term mortality has been linked to a lower PaCO_2_ level at admission (39). Therefore, maintaining appropriately low levels of CO2 partial pressure is crucial for HF patients.

The findings of this study’s binary logistic regression analysis further showed that, in HF patients, RHF is an independent related factor of COPD. One well-researched COPD consequence is RHF. Pulmonary hypertension (PH) develops in COPD patients as a result of systemic inflammation and chronic hypoxia. PH leads to right ventricular hypertrophy, dilatation and systolic dysfunction (8). Compared to having COPD alone, the combination of COPD and PH is associated with a 70% higher risk of death (40).

The strength of the current study lies in its dual focus on clinical characteristics and objective blood markers to evaluate factors associated with COPD in HF patients. This comprehensive approach is seldom addressed in studies involving the Chinese population. However, it should be noted that this study is subject to several limitations. First, being a cross-sectional study, the results should only be interpreted as an association rather than causal relationships between COPD and the selected variables. Secondly, the absence of lung function data necessitated the identification of COPD patients based solely on their medical history, which could introduce information bias. Considering that COPD is a multifactorial disease with lung function being a crucial diagnostic criterion, the lack of comprehensive lung function data in our study restricts the depth and precision of our analysis. Finally, in addition to smoking habits, factors like regional air pollution, indoor fuel use in rural areas, malnutrition in certain regions, and a family history of respiratory diseases significantly impact COPD prevalence. These factors, along with disease severity and age stratification, differ from those in Western or urban populations. Our study, however, lacks detailed analyses in these critical areas.

## 5 Conclusion

In conclusion, a sizable sample of Chinese HF patients in this study had a significant prevalence of COPD. It was observed that patients with both COPD and HF exhibited lower SBP, reduced triglyceride levels, and elevated PaCO_2_. Furthermore, it was found that among patients with HF, male sex, age, a greater ACCI, and right heart failure were independent predictors of COPD. Therefore, when treating patients with heart failure, it is important to monitor and manage their blood pressure, address comorbidities, ensure adequate nutritional support, and optimize carbon dioxide excretion to mitigate the risk of developing COPD.

## Data Availability

Publicly available datasets were analyzed in this study. This data can be found here: Zhang et al. (15), available at https://doi.org/10.13026/8a9e-w734.
